# New information on *Brindabellaspis stensioi* Young, 1980, highlights morphological disparity in Early Devonian placoderms

**DOI:** 10.1098/rsos.180094

**Published:** 2018-06-20

**Authors:** Benedict King, Gavin C. Young, John A. Long

**Affiliations:** 1 College of Science and Engineering, Flinders University, PO Box 2100, Adelaide, South Australia 5001, Australia; 2 Naturalis Biodiversity Center, Postbus 9517, 2300 RA Leiden, The Netherlands; 3 Research School of Physics and Engineering (RSPE), Australian National University, Canberra 0200, Australian Capital Territory, Australia; 4 Australian Museum Research Institute, 1 Williams Street, Sydney New South Wales 2010, Australia

**Keywords:** placoderms, Devonian, lateral line

## Abstract

Acid-prepared specimens of the placoderm *Brindabellaspis stensioi* (Early Devonian of New South Wales, Australia) revealed placoderm endocranial anatomy in unprecedented detail. *Brindabellaspis* has become a key taxon in discussions of early gnathostome phylogeny, and the question of placoderm monophyly versus paraphyly. The anterior orientation of the facial nerve and related hyoid arch structures in this taxon resemble fossil osteostracans (jawless vertebrates) rather than other early gnathostomes. New specimens of *Brindabellaspis* now reveal the previously unknown anterior region of the skull, including an exceptionally elongate premedian bone forming a long rostrum, supported by a thin extension of the postethmo-occipital unit of the braincase. Lateral overlap surfaces indicate an unusual anterior position for the jaws. Digital rendering of a synchrotron radiation scan reveals a uniquely specialized ethmoid commissure sensory canal, doubled back and fused into a midline canal. The visceral surface of the premedian bone has a plexus of perichondral bone canals. An updated skull roof reconstruction of *Brindabellaspis* adds to the highly variable dermal skull patterns of the probably non-monophyletic ‘acanthothoracids'. The unusual morphology revealed by the new specimens suggests that the earliest known reef fish fauna contained a diverse range of fishes with specialized ecological roles.

## 1. Introduction

The Pragian–Emsian Wee Jasper–Lake Burrinjuck limestones preserve the oldest known tropical reef gnathostome assemblage, which includes over 70 species of fossil fishes [1]. The placoderms were the dominant group in this fauna, with at least 45 species assigned to five major placoderm subgroups: arthrodires, ‘acanthothoracids’, petalichthyids, ptyctodontids and a rhenanid. One of the most significant discoveries from the fauna was the placoderm *Brindabellaspis stensioi* Young, 1980 [2], which revealed endocranial anatomy in unprecedented detail.

*Brindabellaspis* has variably been assigned to or compared with the ‘acanthothoracids' (see §2), a poorly defined placoderm assemblage sharing characters including dorsal nasal capsules, a premedian plate and a short trunk armour. However, all of these characters are also known in other placoderm groups [3], and the monophyly of ‘acanthothoracids’ has been questioned [4,5].

‘Acanthothoracids', particularly *Brindabellaspis,* are of special interest in the study of gnathostome phylogenetics, specifically the question of whether placoderms form a paraphyletic grade [6–12] or a clade [13,14]. Endocranial anatomy of non-arthrodire placoderms was initially best known for *Brindabellaspis* [2] and *Macropetalichthys* [15,16]. More recently, digital segmentation studies have produced detailed knowledge of the ‘acanthothoracid’ *Romundina* [17], and new information on the petalichthyid *Shearsbyaspis* [18]*.* Arguments for and against placoderm paraphyly hinge, in large part, on comparisons of this small number of taxa with jawless osteostracans and galeaspids on the one hand, and arthrodires and crown gnathostomes on the other [6,7,10,14,18].

In analyses that have recovered placoderm paraphyly, the precise branching order of placoderms has varied, but arthrodires have been consistently placed crownwards of petalichthyids, antiarchs and *Brindabellaspis*. Originally *Brindabellaspis* was placed crownwards of antiarchs, in a polytomy with petalichthyids and other gnathostomes [6,8–10,12]. More recently, *Brindabellaspis* has been recovered in a polytomy with antiarchs and other gnathostomes [11,19]. When *Brindabellaspis* and the ‘acanthothoracid' *Romundina* are included in the same analysis, they never form a clade, with *Romundina* always more crownward on the gnathostome stem than *Brindabellaspis* [10–12,19].

The status of ‘acanthothoracids' has important implications for the study of gnathostome evolution. ‘Acanthothoracid' paraphyly suggests that some of their features represent the ancestral condition for gnathostomes. For example, the structure of the cranial cavity of *Brindabellaspis* has been compared to jawless vertebrates [5], as has the facial morphology of ‘acanthothoracids', with their dorsal nasal capsules and an elongate trabecular region [10,20]. This facial morphology has been considered an intermediate condition between jawless and jawed vertebrate cranial anatomies [10].

A potentially confounding factor for resolving the phylogenetic position of *Brindabellaspis* is its highly unusual morphology compared with other placoderms. The nasal capsules are situated within the anterior cavity of the orbits, the braincase is unusually deep, and the hyoid arch attachment has an extreme anterior position, suggesting that the jaws of *Brindabellaspis* (unknown) were situated largely anterior to the orbits [2]. Any new information on the previously unknown anterior part of the skull is therefore important for reassessing the phylogenetic significance of *Brindabellaspis*.

Here, we present descriptions of new specimens that reveal the hitherto unknown rostral region of *Brindabellaspis,* and show that its morphology was even more unusual than initially interpreted*.* The pattern of dermal skull roof bones anterior to the orbits is described in detail for the first time, and we apply synchrotron X-ray tomography to investigate a unique specialization of the lateral line sensory system. The highly specialized morphology of *Brindabellaspis* expands the known morphological disparity of early placoderms, and shows that placoderms filled a more diverse array of ecological niches in the Early Devonian than previously thought.

## 2. Systematic palaeontology

Class PLACODERMI McCoy, 1848 [21]

Order BRINDABELLASPIDA Gardiner, 1993 [22]

Family BRINDABELLASPIDAE Gardiner, 1993 [22]

**Remarks**— *Brindabellaspis* has variously been assigned to ‘acanthothoracids’ and ‘rhenanids', two putative groups of placoderms with complex histories. The order Acanthothoraci of Stensiö 1944 [23] originally included the genera *Palaeacanthaspis* and *Dobrowlania* in the family Palaeacanthaspidae Stensiö 1944 [23]. Other ‘acanthothoracids' described prior to the publication of *Brindabellaspis* were *Kolymaspis* [24], *Kosoraspis* [25], *Kimaspis* [26], *Radotina* [27] and *Romundina* [28]. The order Rhenanida of Broili 1930 [29] originally included *Asterosteus, Gemuendina* and *Jagorina*, three flattened ray-like placoderms with enlarged pectoral fins and dorsal eyes and nares.

Most authors have used general resemblances, including dorsal nares, to compare or list *Brindabellaspis* with the order Acanthothoraci (e.g. [1,3,5,30,31]). However, Gardiner [22] recognized its distinctive morphology by erecting a new order and family for this genus. We provisionally follow that treatment here, given that ‘acanthothoracids’ have not been recovered as monophyletic in various recent phylogenies [10–12,14,19].

Young [2] originally placed *Brindabellaspis* within a broader ‘rhenanid' grouping defined by dorsal nasal openings. Included were the Rhenanida *sensu stricto* of Broili [29], plus the various ‘palaeacanthaspid' genera listed above. These were all united by having nares in a mid-dorsal position, compared with the more lateral position in *Brindabellaspis*. Embryological evidence from living groups was cited to support a ventral position for nasal openings being the primitive condition ([2], p. 54). Denison [32] grouped the above ‘palaeacanthaspids' in the order Acanthothoraci and family Palaeacanthaspidae of Stensiö 1944 [23]. Most subsequent authors have followed this usage, the informal ‘acanthothoracid' replacing ‘palaeacanthaspid'. Similarly, Denison [32] included the above ray-like placoderms in the order Rhenanida of Broili [29], also generally followed by subsequent authors, the informal ‘rhenanid’ replacing ‘gemuendinid' of previous usage. Denison [32] considered the dorsal nares of rhenanids to be acquired independently of acanthothoracids.

In the same year, White [33] described a new genus *Weejasperaspis* from the Burrinjuck fish assemblage, placed in its own family within the order Acanthothoraci. Young [2] suggested that *Brindabellaspis* might be closely related to *Weejasperaspis* on the evidence of trunk-shield morphology. Long [31] erected a third Australian ‘acanthothoracid’ genus, *Murrindalaspis*, also placed in the family Weejasperaspidae [33] on the evidence of two similarities: the ornament, and a crest on the median dorsal plate. As the skull of *Murrindalaspis* and the median dorsal plate of *Brindabellaspis* were both unknown, whether one or the other might be closer to *Weejasperaspis* could not be determined on available evidence [31].

Later genera assigned to ‘Acanthothoraci' include *Breizosteus* Goujet 1980 [34], *Connemarraspis* Burrow 2006 [35], *Hagiangella* Dupret *et al*. 2011 [36] and *Arabosteus* Olive *et al*. 2011 [37]. Only *Arabosteus* is represented by skull and braincase material that can be compared with *Brindabellaspis*, and it was assigned to the family Palaeacanthaspidae [23] on the basis that this was the only family (but Weejasperaspidae of White [33] was overlooked). Similarly, Early Devonian forms from the Prague Basin have been assigned to the order Acanthothoraci and family Palaeacanthaspidae [38], although previously there have been reservations [33] that *Radotina* and associated forms belong to ‘typical' acanthothoracids.

Burrow ([35], pp. 61–62) modified Denison's [32] diagnosis of the order Acanthothoraci using characters from Goujet & Young [39]. Of various recent publications that describe or analyse ‘acanthothoracids', only one provides an updated diagnosis [37]. Apart from three features (deep posterior skull embayment bounded by strongly projecting paranuchals; some skull bones separated or overlain by tesserae; ornamental tubercles commonly stellate) *Brindabellaspis* conforms to that diagnosis. The ornament of *Arabosteus* was noted to differ from typical acanthothoracids, and resemble *Brindabellaspis*, in lacking stellate tuberculation, but its other morphological features were considered to indicate provisional assignment to the family Palaeacanthaspidae, rather than Brindabellaspidae [22]. Thus, the order Brindabellaspida and family Brindabellaspidae at present contains only a single genus and species.

Genus *BRINDABELLASPIS* Young, 1980 [2]

*BRINDABELLASPIS STENSIOI* Young, 1980 [2]

**Type skull material**—Two specimens were described by Young [2]: the holotype (**ANU V1677**), and another eroded skull revealing much of the endocranial cavity (**ANU V1678**).

**Figure 1.**
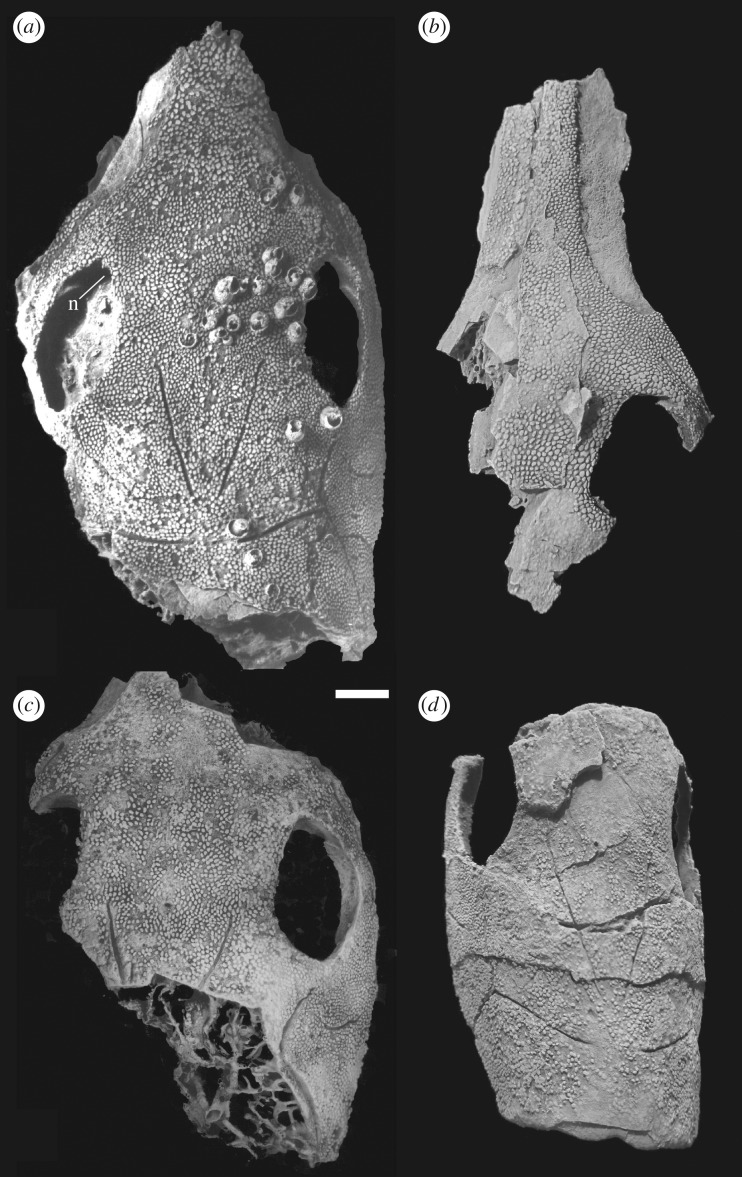
New specimens of *Brindabellaspis stensioi*. (*a*) AM F81911. (*b*) ANU V1224. (*c*) ANU 49493. (*d*) ANU V2584. All dorsal view. Scale bar 10 mm.

**Figure 2.**
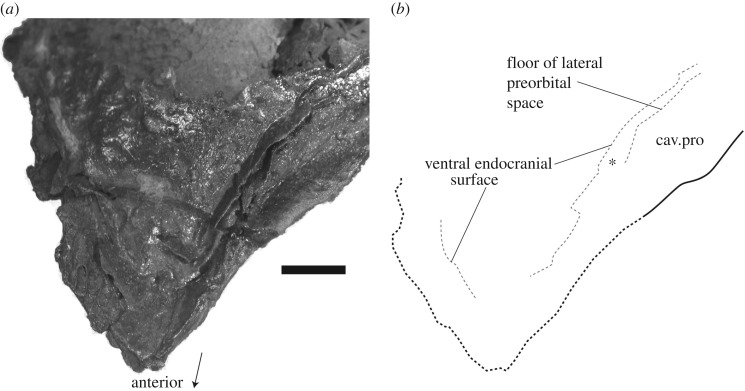
A thin layer of cartilage underlay the premedian plate in *Brindabellaspis.* (*a*) Ventral view of AM F81911, which preserves some of the ventral endocranial surface and floor of the lateral preorbital space beneath the premedian plate. The cartilage below the anterior part of the plate was evidently very thin. Asterisk indicates a thicker region of cartilage just anterior to the lateral preorbital space, where a palatoquadrate attachment is tentatively restored in figure 4*d*. (*b*) Interpretive drawing of (*a*), broken margins dotted in. Perichondral bone in grey, dermal in black. Scale bar represents 10 mm.

**Figure 3.**
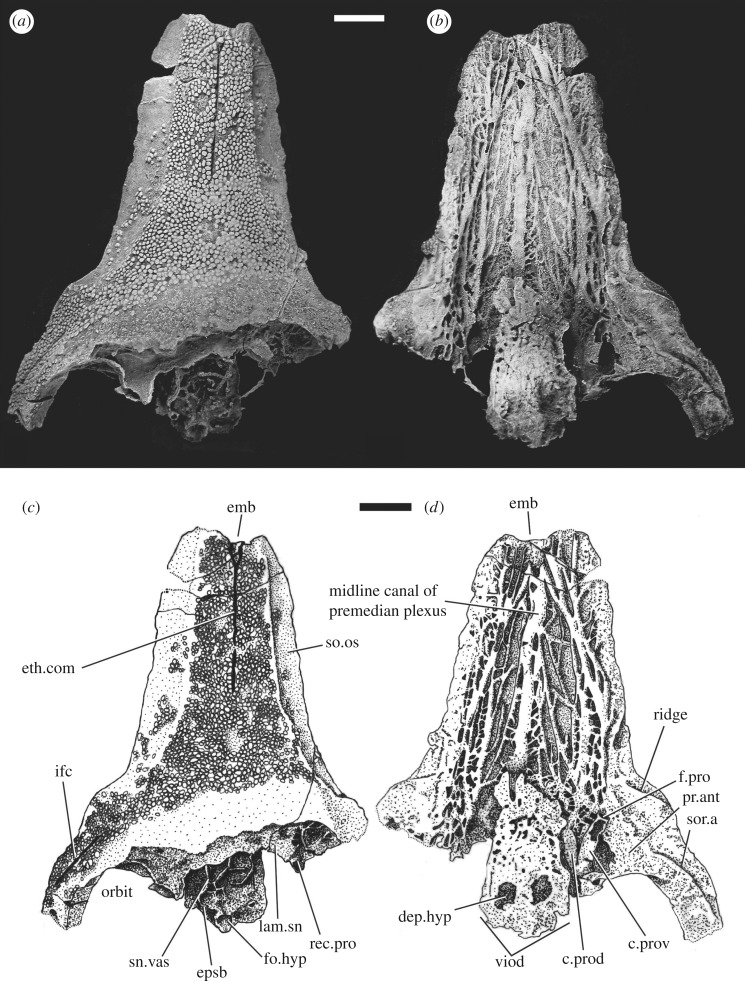
The elongate premedian plate of *Brindabellaspis.* ANU V3247 in dorsal (*a*) and ventral (*b*) views. (*c,d*) Interpretative drawings of *a* and *b*. Scale bars represent 10 mm.

**New skull material**—Five new *Brindabellaspis* specimens provide additional evidence on skull morphology. **AM F81911** (figures 1*a* and 2) is a partial skull and braincase, partly acid-etched from limestone, which was the basis for the skull reconstruction of Young [13]; **ANU V1224** (figure 1*b*) is an incomplete fractured anterior portion of the skull roof showing much of the premedian plate and part of the right orbit; **ANU 49493** (figure 1*c*) is a completely acid-etched partial skull and braincase, its left lateral view previously figured by Goujet & Young [3]; **ANU V2584** (figure 1*d*) is an incomplete fractured and distorted skull that shows a complete posterior margin; **ANU V3247** (figure 3) is a slightly distorted premedian plate with underlying perichondral ossifications, broken off posteriorly at the anterior edge of the orbit and nasal cavity.

**Figure 4.**
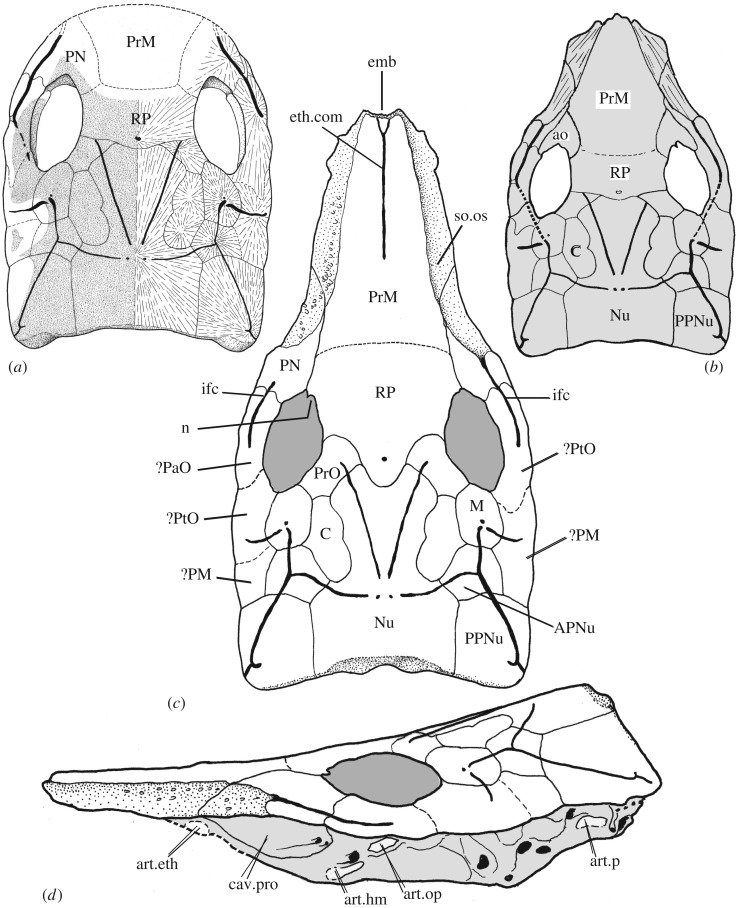
Reconstructions of the pattern of dermal skull roof bones in *Brindabellaspis*. (*a*) Based on Young ([2], fig. 1a). (*b*) After Young ([13], fig. 4h), based on AM F81911. (*c*) Provisional new interpretation. Relative proportions of the preorbital region based on ANU V3247; posterior margin after ANU V2584; nasal notches in the left and right orbit are as developed in AM F81911. (*d*) Lateral reconstruction.

## 3. Material and methods

The type material of Young [2], and the new specimens listed above, have been partly (AM F81911, ANU V1224) or completely (ANU 49493, V2584, V3247) removed from the limestone matrix by etching in dilute acetic or formic acid, the bone strengthened with Mowital or Paraloid during extraction.

ANU V3247 was scanned at the imaging and medical beam of the Australian Synchrotron facility in Melbourne. A total of 26 overlapping 3 mm subscans were imaged, covering an oblique longitudinal strip from the right anterior to the left posterior of the specimen. It was scanned with a monochromatic beam at 30 keV. Sample to detector distance was 325 mm. Pixel size was 6.122 µm and an nRuby detector was used. A total of 1800 projections over 180° were taken. The raw data were processed using the X-TRACT software. The images from the overlapping 3 mm subscans were concatenated for segmentation. Three-dimensional segmenting of the bone and internal canals was performed using Mimics 17.0.

Institutional abbreviations—AM, Australian Museum, Sydney, Australia; ANU, Australian National University, Canberra, Australia; NHMUK, Natural History Museum, London, UK.

Anatomical abbreviations—**ao**, antorbital plate; **APNu**, anterior paranuchal plate; **art.eth**, ethmoidal articulation for palatoquadrate; **art.hm**, hyomandibular articulation; **art.op**, opercular articulation; **art.p**, articulation for posterior pharyngobranchial; **C**, central plate; **cav.pro**, lateral preorbital space; **c.prod**, dorsomesial branch of preorbital canal; **c.prov**, ventral branch of preorbital canal; **dep.hyp**, hypophyseal depression; **emb**, median embayment on anterior margin of premedian plate; **epsb**, canal for the efferent pseudobranchial artery; **eth.com**, ethmoid commissure; **fo.hyp**, hypophyseal fossa; **f.pro**, preorbital foramen; **ifc**, infraorbital sensory canal; **lam.sn**, subnasal lamina; **M**, marginal plate; **n**, notch in orbital margin possibly for nasal opening; **Nu**, nuchal plate; **PaO**, paraorbital plate; **PM**, postmarginal plate; **PN**, postnasal plate; **PPNu**, posterior paranuchal plate; **pr.ant**, antorbital process of endocranium; **PrM**, premedian plate; **PrO**, preorbital plate; **PtO**, postorbital plate; **RP**, rostropineal plate; **rec.pro**, preorbital recess; **sn.vas**, subnasal vascular plexus; **so.os**, overlap surface for suborbital plate; **sor.a**, anterior suborbital ridge; **viod**, median ventral interorbital depression.

## 4. Results

### 4.1. The premedian plate and underlying neurocranium

AM F81911, ANU 49493, V1224 and V3247 (figures 1 and 3) provide new data to reconstruct the pattern of dermal bones anterior to the orbits. The original skull reconstruction ([2], fig. 1) was based on two specimens with the preorbital part broken away at the same level.

ANU V3247 shows the most complete premedian plate (figure 3), which was greatly elongated, with prominent overlap surfaces (figure 3*c*, so.os) complete on both sides (although slightly asymmetrical due to distortion). These overlap surfaces have a patchy distribution of tubercles. The anterior end is complete on the right side (figure 3). The left side has a small broken portion (due to distortion, the left side is stretched slightly forward), which shows that the anterior margin had a small median embayment (figure 3, emb). The lack of tubercles on the anterior margin and numerous small foramina opening into the median embayment suggest that the anterior end of the rostrum was continued as soft tissue.

The visceral surface of ANU V3247 shows a plexus of perichondrally ossified canals in the basal dermal bone layer (figure 3*b,d*), representing the boundary between the dermal premedian plate above and the cartilage of the endocranium below. An enlarged central canal runs forward in the midline (figure 3*d*), and two large anastomosing lateral branches on each side converge near the anterior end of the central canal.

On the left side of ANU V3247, the dorsal and ventral branches of the preorbital canal are preserved (figure 3*d*, c.prov, c.prod). These were first described by Young ([2], fig. 12), but the continuation of the dorsal branch was previously unknown. ANU V3247 shows the dorsal branch of each side meeting in an anastomosing plexus just beneath the overlying dermal bone, from which the median perichondral canal arises, to run forward to the anterior end of the premedian plate. The dorsal preorbital canal also gives off one large lateral branch (and many smaller anastomosing branches), the main one connecting to the inner lateral branch running forward. Previously [2], it was suggested that the dorsomesial branch of the preorbital canal may be equivalent to the ophthalmicus lateralis canal of *Macropetalichthys* [16], and also recently described in *Shearsbyaspis* [18]. However, because the supraorbital canals in *Brindabellaspis* terminate at the mid-level of the orbits [2], we presume that the superficial ophthalmic nerve did not continue anteriorly through the preorbital canal. Rather, the preorbital canal may have carried branches of the profundus nerve to the premedian plexus.

Distinct foramina in front of and behind the ‘preorbital foramen' of Young [2], where the ventral preorbital canal opens into the lateral preorbital space (preserved on the left side of V3247; figure 3*d*, f.pro), also lead into larger canals joining the lateral anastomosing network. It is presumed these were branches from the structure contained within the ventral preorbital canal. The outer lateral branch arises from a foramen further forward, just inside the dermal groove beneath the posterior end of the suborbital overlap area (figure 3*b,d*).

In *Brindabellaspis*, the endocranium is preserved as a single ossification, although a double perichondral lamina within the endocranium was interpreted as the line of fusion between the rhino-capsular and postethmo-occipital units (Young ([2], fig. 4), lam.sn). This lamina is preserved at the posterior end of ANU V3247 (figure 3*c*, lam.sn), but it is incompletely preserved anteriorly. The synchrotron microtomography scan suggests that anteriorly the lamina breaks up into various (presumably vascular) canals.

A highly unusual aspect of the endocranium of *Brindabellaspis* is its pronounced lateral projection outside the lateral edge of the dermal skull roof, from about the level of the hyomandibular nerve foramen forwards. This was not understood during acid preparation of all the new specimens, so this entire region was broken away. In AM F81911, two broken perichondral laminae extending forward beneath the dermal rostrum are all that remains of this region (figure 2). The upper layer may represent the floor of the lateral preorbital space (figure 2, cav.pro). The upper perichondral lamina attaches to the inner dermal bone surface just anterior to the suture crossing the overlap area on the external surface (figure 2). This attachment may also be preserved as a ridge in ANU V3247 (figure 3*d*, ridge).

Anterior to this, the premedian plate in *Brindabellaspis* is supported only by an anterior expansion of the postethmo-occipital unit of the endocranium, its floor preserved as a single perichondral lamina, and the overlying cartilage supporting the premedian plate evidently reduced to about 3 mm thick anteriorly (figure 2). AM F81911 also shows that the curvature of the ventral surface of the endocranium continued forward beneath the premedian plate, following the curvature of the overlying dermal bone.

### 4.2. Ethmoid commissure

The premedian plate bears a median sensory line canal (preserved in AM F81911, ANU V1224, V3247), the last specimen the only one showing its forked anterior end (figure 3*c*, eth.com). Paired foramina, clearly visible in anterior view on the anterior margin, may indicate continuation of these sensory canals into the soft tissue of the rostrum.

**Figure 5.**
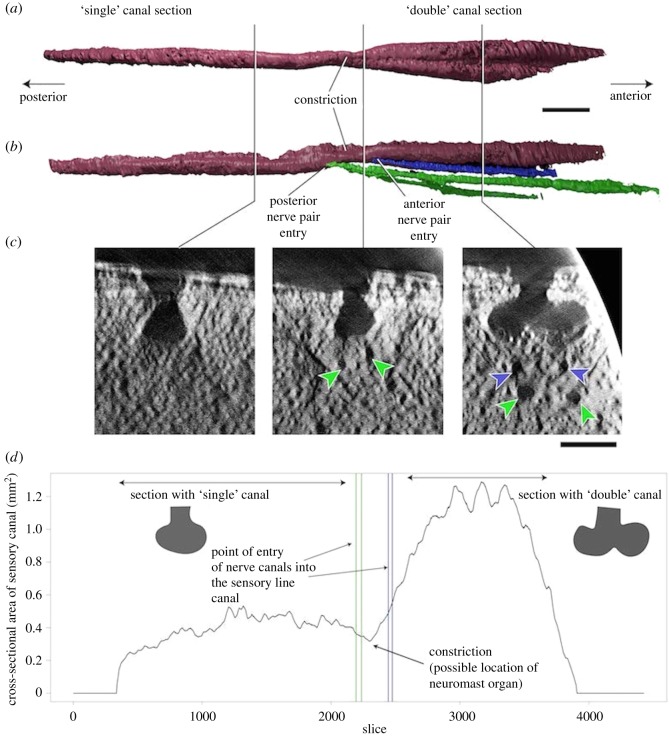
Ethmoid commissure of *Brindabellaspis*. (*a*) Three-dimensional rendering of the canal in Mimics, ventral view. (*b*) Right lateral view of the ethmoid commissure and associated nerve canals. (*c*) Computed tomography slices from three different points along the ethmoid commissure, showing the transition from ‘double’ morphology anteriorly to ‘single' morphology posteriorly. Arrowheads indicate the nerve canals shown in part (*b*). (*d*) Cross-sectional area of the ethmoid commissure along the anteroposterior axis, showing constriction at the point of nerve canal entry. Scale bars represent 2 mm (*a,b*) and 1 mm (*c*).

ANU V3247 shows that the median sensory canal is connected to the perichondral plexus on the ventral surface of the plate via two pairs of canals (figure 5*b*), presumed to carry nerves. They run in a posterodorsal direction from the large midline canal in the perichondral plexus below the premedian plate, and connect to the sensory line at a slight constriction (figure 5*b*). At this same point, the cross-section of the sensory line changes from being obviously double anteriorly to a single fused sensory line posteriorly (figure 5*a*). Individual synchrotron microtomography slices at three points (figure 5*c*) show the transition from a double canal anteriorly to a fused single canal posteriorly. The cross-sectional area of the ethmoid commissure mask in Mimics, plotted along the anterior–posterior axis (figure 5*d*), shows that the anterior section (with the double canal morphology) has a much larger cross-sectional area than the posterior section (with the single canal morphology). It also clearly shows a constriction at the transition between the ‘double' and ‘single' sections.

### 4.3. Dermal skull roof reconstruction

Bone sutures are generally not readily distinguishable in any skull specimen of *Brindabellaspis*, so our new interpretation (figure 4) remains provisional. The preorbital area is of key interest, being completely unpreserved in the original material. The long rostrum, with extensive overlap areas on each side, is based mainly on ANU V3247.

For the post-pineal part of the skull, the new reconstruction generally follows that of Young [2]. This was based on radiographs, radiating striations on the inner surface of the holotype and assumptions about sensory grooves passing through ossification centres of skull bones (right side, figure 4*a*). However, ornament alignment in AM F81911 and ANU 49493 (figure 1*a,c*) suggests that the suture behind the pineal opening may be more V-shaped than first reconstructed. This region was previously only known from one abraded specimen (the holotype). The interpretation of bone sutures lateral and posterolateral to the orbits remains very uncertain, and alternatives as previously discussed [2] are shown on the left and right sides of figure 4*c*. No specimen shows evidence of an anterior suture separating the pineal from the rostral plate, so we interpret a composite rostropineal in this position.

Small bones (figure 4*b*, ao) were previously interpreted anterior to the orbits [13], based on slightly raised areas delimited by notches in the orbital margin in AM F81911 (figure 1*a*, n). These bones do not have an obvious equivalent in other placoderms, and if present would be apomorphic for *Brindabellaspis*. However, both sides of ANU 49493 lack this elevated area of ornament. The right side of ANU V1224 shows a slight elevation similar to AM F81911. The new evidence of these specimens thus suggests that the raised ornamented area in AM F81911, variably developed or absent in other examples of *Brindabellaspis*, does not delimit a separate bone, but is more likely to be equivalent to elevations anterior to the orbits seen in some other placoderms, for example the petalichthyid *Shearsbyaspis* [18,40].

The left side of ANU 49493 shows traces of a suture crossing the overlap area in front of the orbit, and this suture is clearly visible on both sides of AM F81911 (figure 1*a,c*). A notch and vertical partition in AM F81911 may represent the posterior end of this suture.

A ‘postnasal' element around the anterior margin of the orbit (figure 4*a,c*, PN), as previously interpreted [2], would be anterior to the inferred position of nasal openings in *Brindabellaspis*. Good evidence for a separate postnasal element is provided by the clear overlap area for its posterior suture lateral to the orbit as preserved on the right side of ANU V1678 ([2], pl. 1, fig. 4), and a ridge is preserved in this position in the new specimens (figure 1*a–c*). In AM F81911, the infraorbital sensory groove passes onto the suborbital overlap area on both sides (figure 1*a*), but in ANU V3247 it terminates well behind the overlap (figure 3). This variation is shown on the left and right sides of the reconstruction (figure 4*c*).

The postnasal element can be assumed to connect mesially with the unpaired rostropineal plate, and thus including slight notches in the anterodorsal corner of the orbits in AM F81911 (figure 1*a*, n), previously interpreted to represent a nasal notch ([13], fig. 4h). The left side of AM F81911 suggests a connecting groove to the nasal cavity, which may have carried a nasal tube. This part of the orbital margin was completely unknown in the original material (badly abraded in the holotype; missing in ANU V1678). A distinct process in the left orbit of AM F81911 (less developed on the right side) delineates a separate anterior notch, now considered to be the end of the bone suture described above. The anterodorsal margin of the orbit is otherwise preserved only in ANU 49493 (both sides) and ANU V1224 (right side), where these slight notches are less distinct and variably developed.

An anterior suture to the rostropineal element, separating it from the unpaired premedian plate, is assumed by comparison with other placoderms possessing this bone. There is no indication of this suture in the new specimens, so the posterior extent of the premedian plate remains very uncertain. It can be assumed that the suture was anterior to the nasal notches, and posterior to the median sensory groove (ethmoid commissure), which we assume was confined to the premedian plate, as in other placoderms where present. Given the great rostral elongation, the rostropineal bone is shown somewhat larger than in the original reconstruction, on the assumption that it was also elongated to some extent. A comparison with the elongate rostrum in some arthrodires shows that elongation of the rostral bone also involves the pineal and preorbital bones (*Carolowilhelmina* [41]), elongate preorbitals only (*Rolfosteus* [42]) or an elongate postnasal element (*Tubonasus* [42]).

## 5. Discussion

### 5.1. Comparison with previous interpretations of *Brindabellaspis*

The new specimens of *Brindabellaspis* provide information on the anterior part of the skull, demonstrating the presence of an elongate premedian plate, and the dermal bone pattern anterior to the orbits (figure 4). The most recent previous skull reconstruction of *Brindabellaspis* came from a review paper [13] based on a preliminary study of AM F81911 for the preorbital region. The five new specimens now indicate a quite different dermal bone pattern, comprising elongate rostropineal and premedian bones in the midline and paired postnasal elements anterolateral to the orbits.

The suborbital plate and palatoquadrate remain unknown in *Brindabellaspis,* but the available material provides some clues about their configuration. The large overlap areas on either side of the rostrum are interpreted as for the suborbital plate. However, given that the overlap surface is partly covered in tubercles, it is possible that the overlaps were partly covered in tesserae (known from the skulls of various petalichthyid and ‘acanthothoracid’ placoderms [38,43,44]), and that the suborbital plate did not extend the full length of the premedian plate. The palatoquadrate attachments are unknown, but the cartilage underlying the anterior part of the premedian plate was very thin (figure 2), possibly too thin to support an articulation. It was thicker further posteriorly, just anterior to the lateral preorbital space (figure 2, asterisk), and so a palatoquadrate attachment may have been in this region. A new lateral braincase restoration shows a single ethmoid articulation (figure 4*d*, art.eth) in this position, although we note that in *Romundina* three palatoquadrate attachment areas are present on the endocranium lateral to the premedian plate [10,28], even though this bone is much shorter than in *Brindabellaspis*.

Three other articular surfaces are known on the lateral braincase surface of *Brindabellaspis*, the most posterior (figure 4*d*, art.p) probably for a gill arch, and another (art.op) interpreted as for an opercular cartilage [2]. The hyoid arch articulation (art.hy), as previously interpreted [2], lies anteroventral to the hyomandibular nerve opening, in an unusually anterior position. Gardiner [30] preferred to interpret this as a palatoquadrate articulation, and the more posterior articulation (the opercular cartilage articulation of Young) as the hyomandibular articulation. However, new evidence from early arthrodires [45], and *Romundina* [17], demonstrates separate opercular and hyomandibular articulations associated with the hyomandibular nerve foramen, and no contact with the palatoquadrate.

### 5.2. Comparisons with other placoderms

As interpreted (figure 4*c*), the skull roof pattern of *Brindabellaspis* is unique. The large rostropineal plate is sutured firmly to the rest of the skull roof, which shows unique rostral elongation resulting from its long premedian plate. This places the centre of the orbits in the posterior half of the skull roof length (about 42% of skull length from the posterior margin). Other placoderms with orbits enclosed in the skull roof have orbits in a more anterior position, even when a pronounced rostrum is developed. Thus, in *Wuttagoonaspis* (which lacks a premedian plate) the orbits are 33–45% of skull length from the anterior margin [46], and in petalichthyids (e.g. *Macropetalichthys*) this is about 30% (accentuated by the nuchal region being more elongate in petalichthyids than in *Wuttagoonaspis* or *Brindabellaspis*). Also unique is the position of the nasal capsules within the orbits, in contrast with other acanthothoracids (as represented by *Romundina*) where the nasal openings occupy the space between the rostral capsule and the premedian plate [10,17,47].

The unusual skull roof pattern is also shown in the position of the postnasal element, around the anterior margin of the orbit. This would be anterior to the nasal openings of *Brindabellaspis*, presumably located within the orbits as previously interpreted [2]. In *Radotina*, a postnasal element has been restored lateral to the nasal opening [44], but mesial to the orbit, whereas in *Brindabellaspis* the equivalent element is lateral and anterolateral to the orbit. The postnasal element shows notches in its orbital margin in some specimens. If representing nasal openings, this would be comparable to the notched postnasal of brachythoracid arthrodires (e.g. [48]). Possibly the nasal opening was bounded laterally by a dermal process of the sclerotic capsule (unknown for *Brindabellaspis*), as in the isolated weejasperaspid sclerotic capsule previously described [49]. This structure is also seen in early brachythoracids [45] and other more distantly related placoderm taxa such as antiarchs [50] and *Entelognathus* [9]. Possibly both incurrent and excurrent nasal openings were dorsal in position for *Brindabellaspis*, because the floor of the orbit and preorbital space probably occluded any ventral passage.

The braincase of *Brindabellaspis* may be compared with that of *Romundina,* and the proportions differ greatly due to the rostral elongation in *Brindabellaspis.* As previously shown [2], the division of the braincase into separately ossified postethmo-occipital and rhinocapsular units is still evident in *Brindabellaspis,* with these units fused together and the division represented by a double perichondral lamina (the subnasal lamina; figure 3*a,c*, lam.sn) within the braincase. The skull roof is also consolidated, showing no trace of division into a separate rostral capsule, and the nasal capsules are in a more lateral position within the orbits. In *Romundina*, the rhinocapsular unit is separately ossified, and posteriorly placed between the orbits. The postethmo-occipital unit (specifically the trabecular region) extends anteriorly and underlies the premedian plate [10]. In *Brindabellaspis*, the subnasal lamina evidently terminated adjacent to the posterior end of the premedian plate, and the entire premedian plate is underlain by a thin extension of the postethmo-occipital part of the braincase. Thus, the trabecular region in *Brindabellaspis* would have an even larger preorbital extension than in *Romundina*.

Clearly, the morphology of *Brindabellaspis* is quite different from that of other ‘acanthothoracids'. Dermal skull roof patterns were already recorded to be highly variable within that assemblage, sometimes including zones of tesserae between the larger bones [44]. Dermal bone patterns have also been found to vary intraspecifically [37], although similar variation in ‘*Radotina'* has been explained by previously unknown species diversity [38]. This variability in dermal bone pattern might be taken as additional evidence that ‘acanthothoracids' may not be monophyletic, although dermal skull roof characters are often difficult to polarize.

Comparing the morphology of *Brindabellaspis* with that of *Romundina*, obvious differences can be explained by two possibly related changes: great rostral elongation of the ethmoid region and the consolidation of the rostral capsule with the rest of the skull. In *Romundina*, the nasal capsules occupy the space between the rostral capsule and the premedian plate. However, in *Brindabellaspis*, the rostral is firmly fused to the premedian, which would be possible only with a more lateral position for the nasal capsules within the orbits. Similarly the comparatively long premedian plate, the extensive underlying trabecular region and the anterior position of the hyoid arch attachment are all likely to be related: these features have essentially the same relative positions as in *Romundina,* but the whole ethmoid region is stretched anteriorly in comparison.

Another similarity with *Romundina* is the plexus of perichondral canals underlying the premedian plate. Possibly such a plexus was a common feature of all placoderms with a premedian plate, with the lack of braincase ossification obscuring its presence in antiarchs. The base of the premedian plate in the antiarch *Bothriolepis* shows significant porosity ([51], pl. 57), which could be related to a similar plexus. In both *Romundina* and *Brindabellaspis*, the plexus comprises larger canals running forward in the midline, and converging anteromesially from both sides ([47], fig. 2B2). However, it is difficult to say on present evidence whether these similarities could be characters linking all ‘acanthothoracids’, due to the absence of an equivalent degree of preservation in most other placoderms.

### 5.3. Unique adaptation to the sensory line system

*Brindabellaspis* has a sensory canal on the midline of the premedian plate. An anteroposteriorly directed sensory canal in this position is, to our knowledge, unknown in any other vertebrate. An anterior transverse ethmoid commissure is, however, present in a wide variety of gnathostomes, including on the premedian plate of various ‘acanthothoracid' and antiarch placoderms. The fork at the anterior end of the midline canal in *Brindabellaspis* suggests that this midline canal represents the ethmoid commissure, which has folded back on itself and fused in the midline.

The two pairs of nerve canals that enter the ethmoid commissure either side of the constriction may have innervated a neuromast organ. In some living species, neuromast organs occur at constrictions in the sensory canal [52], which may increase sensitivity by amplifying particle motions within the canal [53]. As there are two pairs of nerve canals in *Brindabellaspis* (shown by ANU V3247), a possible interpretation is that the ethmoid commissure has fused at the point of two neuromasts that would normally be positioned either side of the midline in a transverse ethmoid commissure.

The ethmoid commissure is typically innervated by the buccal nerve in living species [54,55], and in *Romundina* it was suggested that the buccal nerve entered the lateral ends of a transverse neurovascular web underlying the ethmoid commissure [47]. The buccal nerve is associated with the maxillary branch of the trigeminal, which in *Brindabellaspis* ran through the preorbital space*.* It may have entered the perichondral plexus from the anterior to innervate the ethmoid commissure, doubling back to follow the path of the ethmoid commissure itself (electronic supplementary material, figure S1). This would be consistent with the posterodorsal orientation of the nerve canals, which suggests that the nerve fibres within entered the plexus anteriorly.

### 5.4. Ecological role of *Brindabellaspis*

The unusual morphology of *Brindabellaspis* indicates a specialized role, but inferences about the biology of *Brindabellaspis* are somewhat limited without preservation of the jaws. The dorsolaterally positioned eyes of *Brindabellaspis* are consistent with a benthic niche, and the rostrum formed by the premedian plate may have functioned in the detection of bottom-dwelling prey.

The elaborate plexus of perichondral canals underlying the premedian plate might also be suggestive of a sensory function for the elongate rostral region of *Brindabellaspis*. Our current hypothesis is that the tip of the rostrum of *Brindabellaspis* was continued as soft tissue, which could have housed a sensory system. However, the nature of such a sensory system, and the precise functional significance of the perichondral plexus, is unclear. Lungfish also possess a system of perichondral tubes in the cartilage of the snout [56–58], although this forms an upwardly branching system rather than a horizontal plexus. The function of the lungfish tubuli has been a matter of debate, with competing hypotheses that they housed nerves, blood vessels or lymphatics [58–61]. Recently, lungfish rostral tubuli have been suggested to supply nerves to electroreceptors covering the snout [62]. One potential function of the perichondral plexus for *Brindabellaspis* was to supply nerves to electroreceptors on the snout. A possible analogue is the paddlefish *Polyodon,* which uses its rostral paddle as an antenna to seek out plankton, aided by a dense array of electroreceptors on the underside [63]. Similarly, the rostrum of shovelnose rays has dense electroreceptors on the ventral surface for prey detection [64]. Without further evidence, this hypothesis remains largely speculative for *Brindabellaspis*, but possibly the irregular ‘paddle-like' lateral expansion of the rostrum (shown by ANU V1224) may be consistent with that idea.

An alternative is that the plexus played no special functional role in *Brindabellaspis.* In placoderms, such a plexus may be a common feature where endocranial cartilage meets dermal bone over an extended area. For example, the arthrodire *Goodradigbeeon*, also from Burrinjuck, has an extensive internasal wall between the left and right nasal capsules [33]. The roof of the internasal wall, where the cartilage of the rhinocapsular ossification contacts the overlying rostropineal plate, has a plexus of perichondral canals (electronic supplementary material, figure S2). Extensive canal networks are also found at the interface of dermal and perichondral bone throughout the skull roof in *Romundina* [17].

Our new information on *Brindabellaspis* shows that this placoderm, already known to have unusual morphology, was even more specialized than previously thought. There is now good evidence that during the Devonian Period reef ecosystems were, as today, major centres for biodiversity [1,65]. Beyond species diversity, *Brindabellaspis* provides evidence for disparate body forms in the Taemas–Wee Jasper fossil fish fauna, the earliest known example of a diverse coral reef fish assemblage. Disparate body forms are also known from the Late Devonian Gogo Formation, another highly diverse reef assemblage [65]. At Gogo, the long-snouted lungfish *Griphognathus* [57] may have filled a similar ecological niche to that of the placoderm *Brindabellaspis* in the Early Devonian.

## Supplementary Material

Supplement

## Data accessibility

Ply files and a three-dimensional PDF of the digital models are available from the Dryad Digital Repository [66]. Scan data are available via e-researchSA (https://doi.org/10.4226/86/5a05055b47a98).

## References

[1] Young GC 2011 Wee Jasper-Lake Burrinjuck fossil fish sites: scientific background to national heritage nomination. Proc. Linn. Soc. N. S. W. 132, 83–107.

[2] Young GC 1980 A new Early Devonian placoderm from New South Wales, Australia, with a discussion of placoderm phylogeny. Palaeontogr. Abt. A Palaeozool-Stratigr. 167, 10–76.

[3] Goujet D, Young GC 2004 Placoderm anatomy and phylogeny: new insights. In Recent advances in the origin and early radiation of vertebrates (eds Arratia G, Wilson MVH, Cloutier R), pp. 109–126. München, Germany: Verlag Dr. Friedrich Pfeil.

[4] Goujet D 1984 Placoderm interrelationships: a new interpretation, with a short review of placoderm classifications. Proc. Linn. Soc. N. S. W. 201, 211–243.

[5] Janvier P 1996 Early vertebrates. Oxford, UK: Clarendon Press.

[6] Brazeau MD 2009 The braincase and jaws of a Devonian ‘acanthodian’ and modern gnathostome origins. Nature 457, 305–308. (doi:10.1038/nature07436) 19148098 10.1038/nature07436

[7] Brazeau MD, Friedman M 2014 The characters of Palaeozoic jawed vertebrates. Zool. J. Linn. Soc. 170, 779–821. (doi:10.1111/zoj.12111) 25750460 10.1111/zoj.12111 PMC4347021

[8] Davis SP, Finarelli JA, Coates MI 2012 *Acanthodes* and shark-like conditions in the last common ancestor of modern gnathostomes. Nature 486, 247–250. (doi:10.1038/nature11080) 22699617 10.1038/nature11080

[9] Zhu M et al. 2013 A Silurian placoderm with osteichthyan-like marginal jaw bones. Nature. 502, 188–193. (doi:10.1038/nature12617) 24067611 10.1038/nature12617

[10] Dupret V, Sanchez S, Goujet D, Tafforeau P, Ahlberg PE 2014 A primitive placoderm sheds light on the origin of the jawed vertebrate face. Nature 507, 500–503. (doi:10.1038/nature12980) 24522530 10.1038/nature12980

[11] Giles S, Friedman M, Brazeau MD 2015 Osteichthyan-like cranial conditions in an Early Devonian stem gnathostome. Nature 520, 82–85. (doi:10.1038/nature14065) 25581798 10.1038/nature14065 PMC5536226

[12] Long JA et al. 2015 Copulation in antiarch placoderms and the origin of gnathostome internal fertilization. Nature 517, 196–199. (doi:10.1038/nature13825) 25327249 10.1038/nature13825

[13] Young GC 2010 Placoderms (armored fish): dominant vertebrates of the Devonian period. Annu. Rev. Earth Planet. Sci. 38, 523–550. (doi:10.1146/annurev-earth-040809-152507)

[14] King B, Qiao T, Lee MS, Zhu M, Long JA 2017 Bayesian morphological clock methods resurrect placoderm monophyly and reveal rapid early evolution in jawed vertebrates. Syst. Biol. 66, 499–516. 27920231 10.1093/sysbio/syw107

[15] Stensiö EA 1963 The brain and the cranial nerves in fossil, lower craniate vertebrates. Oslo, Norway: Universitetsforlaget.

[16] Stensiö EA 1969 Elasmobranchiomorphi Placodermata Arthrodires. In Traité de paléontologie (ed. Piveteau J), pp. 71–692. Paris, France: Masson.

[17] Dupret V, Sanchez S, Goujet D, Ahlberg P 2017 The internal cranial anatomy of *Romundina stellina* Ørvig, 1975 (Vertebrata, Placodermi, Acanthothoraci) and the origin of jawed vertebrates—anatomical atlas of a primitive gnathostome. PLoS ONE 12, e0171241 (doi:10.1371/journal.pone.0171241) 28170434 10.1371/journal.pone.0171241 PMC5295682

[18] Castiello M, Brazeau MD 2018 Neurocranial anatomy of the petalichthyid placoderm *Shearsbyaspis oepiki* Young revealed by X-ray computed microtomography. Palaeontology 61, 369–389. (doi:10.1111/pala.12345) 29937580 10.1111/pala.12345 PMC5993267

[19] Qiao T, King B, Long JA, Ahlberg PE, Zhu M 2016 Early gnathostome phylogeny revisited: multiple method consensus. PLoS ONE 11, e0163157 (doi:10.1371/journal.pone.0163157) 27649538 10.1371/journal.pone.0163157 PMC5029804

[20] Gross W 1958 Über die älteste Arthrodiren-Gattung. Notizblatt des Hessischen Landesamtes für Bodenforschung zu Wiesbaden. 86, 7–30.

[21] McCoy F 1848 On some new fossil fish of the Carboniferous period. Ann. Mag. Nat. Hist. 2, 1–10. (doi:10.1080/03745485809496133)

[22] Gardiner BG 1993 Placodermi. Fossil Rec. 2, 583–588.

[23] Stensiö EA 1944 Contributions to the knowledge of the vertebrate fauna of the Silurian and Devonian of western Podolia. 2, notes on two arthrodires from the Downtonian of Podolia. Arkiv für Zoologi 35, 1–83.

[24] Buistrow AP 1956 *Kolymaspis sibirica* ng, n. sp., a new representative of the Lower Devonian agnathous vertebrates. Vestn. Leningr. Univ. Geol. Geogr. 18, 5–13.

[25] Gross W 1959 Arthrodiren aus dem Obersilur der Prager Mulde. Palaeontographica Abteilung A 113, 1–35.

[26] Mark-Kurik E 1973 *Kimaspis*, a new palaeacanthaspid from the Early Devonian of Central Asia. Eesti NSV Tead. Akad. Toim. 22, 322–330.

[27] Gross W 1950 Die paläontologische und stratigraphische Bedeutung der Wirbeltierfaunen des Old Red und der marinen altpaläozoischen Schichten. Abh. dt. Akad. Wiss. Berlin Math.-Nat. Kl. 1949, 1–130.

[28] Ørvig T 1975 Description, with special reference to the dermal skeleton, of a new radotinid arthrodire from the Gedinnian of Arctic Canada. Colloq. Int. CNRS 218, 43–71.

[29] Broili F 1930 Über *Gemündina Stürtzi* Traquair. Abh. Bayerischen Akad. Wiss. Mathmatischnaturwissenschaftliche. 6, 1–24.

[30] Gardiner BG 1984 The relationship of placoderms. J. Vertebr. Paleontol. 4, 379–395. (doi:10.1080/02724634.1984.10012017)

[31] Long JA 1984 New placoderm fishes from the Early Devonian Buchan Group, eastern Victoria. Proc. R. Soc. Vic. 96, 173–186.

[32] Denison RH 1978 Placodermi. Stuttgart and New York, NY: Gustav Fischer Verlag.

[33] White EI 1978 The larger arthrodiran fishes from the area of the Burrinjuck Dam, NSW. Trans. Zool. Soc. Lond. 34, 149–262. (doi:10.1111/j.1096-3642.1978.tb00374.x)

[34] Goujet D 1980 Les Poissons. Mém. Soc. Géol. Minéralogique de Bretagne. 23, 305–308.

[35] Burrow CJ 2006 Placoderm fauna from the Connemarra Formation (? late Lochkovian, Early Devonian), central New South Wales. Alcheringa Aust. J. Palaeontol. 30, 59–88. (doi:10.1080/03115510609506856)

[36] Dupret V, Phuong TH, Thanh T-D, Phong ND, Janvier P, Clément G 2011 The skull of *Hagiangella goujeti* Janvier, 2005, a high-crested acanthothoracid (*Vertebrata, Placodermi*) from the Lower Devonian of northern Vietnam. J. Vertebr. Paleontol. 31, 531–538. (doi:10.1080/02724634.2011.558148)

[37] Olive S, Goujet D, Lelièvre H, Janjou D 2011 A new Placoderm fish (Acanthothoraci) from the Early Devonian Jauf Formation (Saudi Arabia). Geodiversitas 33, 393–409. (doi:10.5252/g2011n3a1)

[38] Vaškaninová V, Ahlberg PE 2017 Unique diversity of acanthothoracid placoderms (basal jawed vertebrates) in the Early Devonian of the Prague Basin, Czech Republic: a new look at *Radotina* and *Holopetalichthys*. PLoS ONE 12, e0174794 (doi:10.1371/journal.pone.0174794) 28380002 10.1371/journal.pone.0174794 PMC5381876

[39] Goujet D, Young GC 1995 Interrelationships of placoderms revisited. Geobios 28, 89–95. (doi:10.1016/S0016-6995(95)80093-X)

[40] Young GC 1985 Further petalichthyid remains (placoderm fishes, Early Devonian) from the Taemas-Wee Jasper region, New South Wales. Bur. Miner. Resour. J. Aus. Geol. Geophys. 9, 121–131.

[41] Mark-Kurik E, Carls P 2002 A long-snouted Late Eifelian arthrodire from Aragón, Spain. Rev. Esp. Paleontol. 17, 117–135.

[42] Dennis K, Miles RS 1979 Eubrachythoracid arthrodires with tubular rostral plates from Gogo, Western Australia. Zool. J. Linn. Soc. 67, 297–328. (doi:10.1111/j.1096-3642.1979.tb01118.x)

[43] Gross W 1961 *Lunaspis broilii* und *Lunaspis heroldi* aus dem Hunsrückschiefer (Unterdevons, Rheinland). Notizblatt Hessisches Landesamtes für Bodenforschung zu Wiesbaden. 89, 17–43.

[44] Westoll TS 1967 *Radotina* and other tesserate fishes. Zool. J. Linn. Soc. 47, 83–98. (doi:10.1111/j.1096-3642.1967.tb01397.x)

[45] Hu Y, Lu J, Young GC 2017 New findings in a 400 million-year-old Devonian placoderm shed light on jaw structure and function in basal gnathostomes. Sci. Rep. 7, 7813 (doi:10.1038/s41598-017-07674-y) 28798392 10.1038/s41598-017-07674-y PMC5552730

[46] Young GC, Goujet D 2003 Devonian fish remains from the Dulcie Sandstone and Cravens Peak Beds, Georgina Basin, central Australia. Rec. West. Aust. Mus. Suppl. 65, 1–80. (doi:10.18195/issn.0313-122x.65.2003.001-085)

[47] Dupret V, Sanchez S, Goujet D, Tafforeau P, Ahlberg PE 2010 Bone vascularization and growth in placoderms (*Vertebrata*): the example of the premedian plate of *Romundina stellina* Ørvig, 1975. C. R. Palevol. 9, 369–375. (doi:10.1016/j.crpv.2010.07.005)

[48] Miles RS, Westoll TS 1968 IX.—the Placoderm fish *Coccosteus cuspidatus* Miller ex Agassiz from the middle old red sandstone of Scotland. Part I. Descriptive morphology. Trans. R. Soc. Edinb. 67, 373–476. (doi:10.1017/S0080456800024078)

[49] Long JA, Young GC 1988 Acanthothoracid remains from the Early Devonian of New South Wales, including a complete sclerotic capsule and pelvic girdle. Mem. Assoc. Australas. Palaeontol. 7, 65–80.

[50] Young GC, Zhang G 1996 New information on the morphology of yunnanolepid antiarchs (placoderm fishes) from the Early Devonian of South China. J. Vertebr. Paleontol. 16, 623–641. (doi:10.1080/02724634.1996.10011353)

[51] Young GC 1984 Reconstruction of the jaws and braincase in the Devonian placoderm fish *Bothriolepis*. Palaeontology 27, 635–661.

[52] Montgomery JC, Saunders AJ 1985 Functional morphology of the piper *Hyporhamphus ihi* with reference to the role of the lateral line in feeding. Proc. R. Soc. Lond. B 224, 197–208. (doi:10.1098/rspb.1985.0029) 2860672 10.1098/rspb.1985.0029

[53] Montgomery JC 1989 Lateral line detection of planktonic prey. In The mechanosensory lateral line (eds Coombs S, Görner P, Münz H), pp. 561–574. New York, NY: Springer.

[54] Jarvik E 1980 Basic structure and evolution of vertebrates. London, UK: Academic Press.

[55] Piotrowski T, Northcutt RG 1996 The cranial nerves of the Senegal Bichir, *Polypterus senegalus* [Osteichthyes: Actinopterygii: Cladistia]. Brain Behav. Evol. 47, 55–102. (doi:10.1159/000113229) 8866706 10.1159/000113229

[56] Thomson KS, Campbell KSW 1971 The structure and relationships of the primitive Devonian lungfish—*Dipnorhynchus sussmilchi* (Etheridge). Bull. Peabody Mus. Nat. Hist. 38, 1–109.

[57] Miles RS 1977 Dipnoan (lungfish) skulls and the relationships of the group: a study based on new species from the Devonian of Australia. Zool. J. Linn. Soc. 61, 1–328. (doi:10.1111/j.1096-3642.1977.tb01031.x)

[58] Cheng H 1989 On the tubuli in Devonian lungfishes. Alcheringa 13, 153–166. (doi:10.1080/03115518908619049)

[59] Campbell KSW, Barwick RE 1986 Paleozoic lungfishes—a review. J. Morphol. Suppl. 1, 93–131. (doi:10.1002/jmor.1051900409)

[60] Bemis WE, Northcutt RG 1992 Skin and blood vessels of the snout of the Australian lungfish, *Neoceratodus forsteri*, and their significance for interpreting the cosmine of Devonian lungfishes. Acta Zool. 73, 115–139. (doi:10.1111/j.1463-6395.1992.tb00956.x)

[61] Kemp A 2014 Skin structure in the snout of the Australian lungfish, *Neoceratodus forsteri* (*Osteichthyes: Dipnoi*). Tissue Cell 46, 397–408. (doi:10.1016/j.tice.2014.07.004) 25175034 10.1016/j.tice.2014.07.004

[62] King B, Hu Y, Long JA 2018 Electroreception in early vertebrates: survey, evidence and new information. Palaeontology 61, 325–328. (doi:10.1111/pala.12346)

[63] Wilkens LA, Russell DF, Pei X, Gurgens C 1997 The paddlefish rostrum functions as an electrosensory antenna in plankton feeding. Proc. R. Soc. Lond. B 264, 1723–1729. (doi:10.1098/rspb.1997.0239)

[64] Wueringer BE, Tibbetts IR 2008 Comparison of the lateral line and ampullary systems of two species of shovelnose ray. Rev. Fish Biol. Fish. 18, 47–64. (doi:10.1007/s11160-007-9063-9)

[65] Long JA, Trinajstic K 2010 The Late Devonian Gogo Formation lägerstatte of Western Australia: exceptional early vertebrate preservation and diversity. Annu. Rev. Earth Planet. Sci. 38, 255–279. (doi:10.1146/annurev-earth-040809-152416)

[66] King B, Young GC, Long JA 2018 Data from: New information on *Brindabellaspis stensioi* Young 1980 highlights morphological disparity in Early Devonian placoderms. Dryad Digital Repository (doi:10.5061/dryad.s353f)

