# Supplementary material for: New information on Brindabellaspis stensioi Young, 1980, highlights morphological disparity in Early Devonian placoderms
Source: R Soc Open Sci. 2018 Jun 20;5(6):180094. doi: 10.1098/rsos.180094 (PMC6030278; doi:10.1098/rsos.180094)
Supplement: Supplement [file rsos180094supp1.pdf]

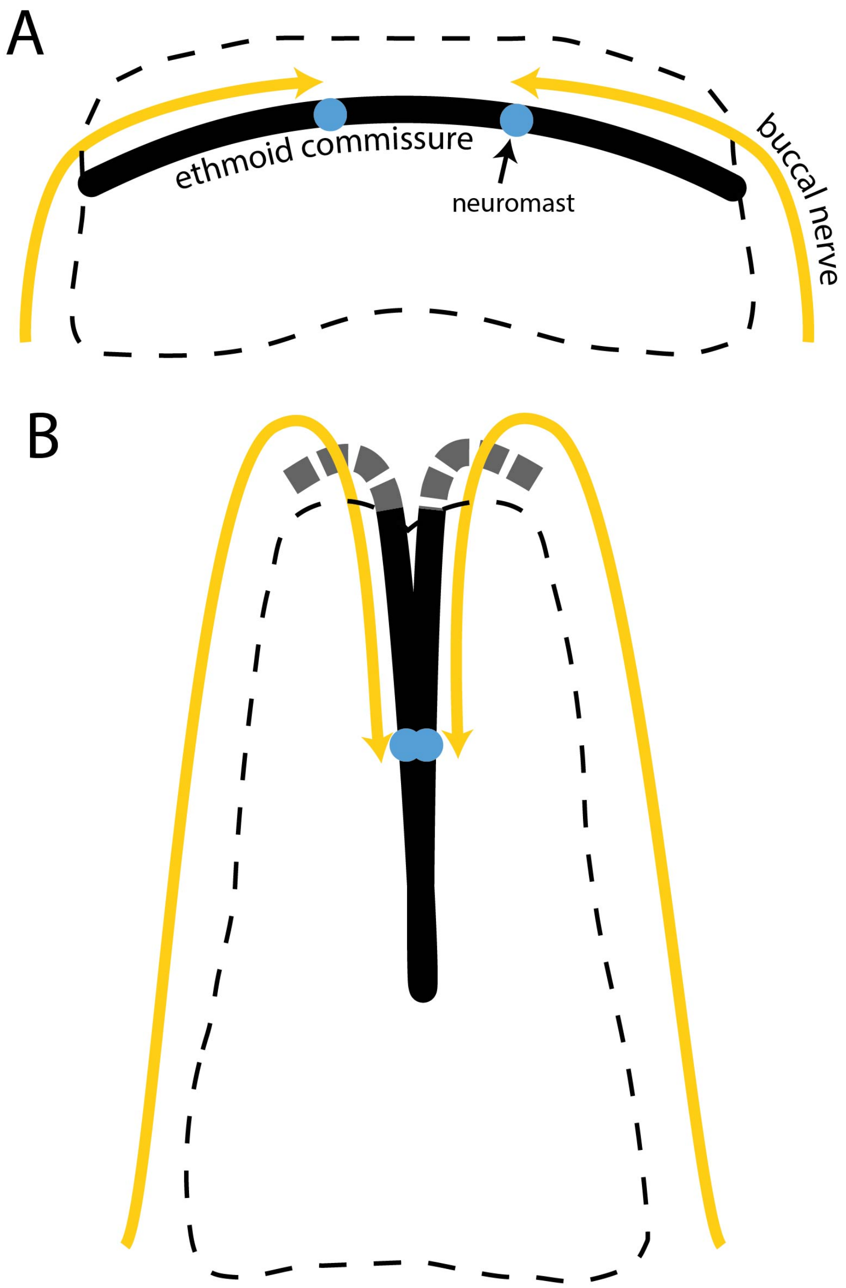

Figure S1. Summary diagram of interpretation of the ethmoid commissure in *Brindabellaspis.* A) Hypothesised ancestral (and empirically more typical) state for the ethmoid commissure, with premedian plate dotted in based on *Romundina*. A transverse canal innervated from either end by the buccal nerve is typical for the ethmoid commissure. B) Interpretation for *Brindabellaspis,* with the ethmoid commissure doubled back into a midline canal and the buccal nerve following the same course. Hypothetical continuation in the soft tissue of the snout dotted in grey.

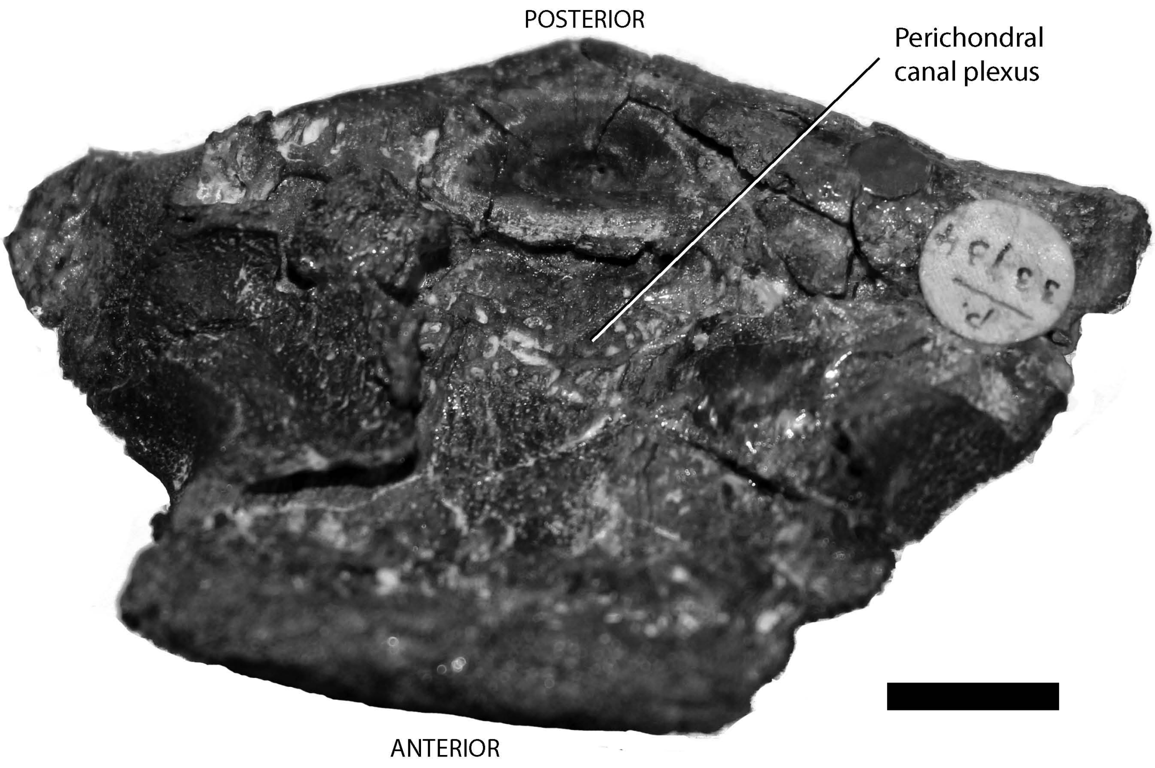

Figure S2. Perichondral canal plexus under the rostral plate of *Goodradigbeeon.* Ventral view of the rostral and pineal plates of *Goodradigbeeon* (NHMUK P33734). A plexus of perichondral canals is apparent where the cartilage of the rhinocapsular ossification would have contacted the overlying rostral plate. Scale bar represents 10mm.
